# Gender-specific differences in central blood pressure and optimal target blood pressure based on the prediction of cardiovascular events

**DOI:** 10.3389/fcvm.2022.990748

**Published:** 2022-10-19

**Authors:** Min-Sik Kim, Gee-Hee Kim

**Affiliations:** ^1^Division of Cardiology, Department of Internal Medicine, St. Vincent's Hospital, College of Medicine, The Catholic University of Korea, Seoul, South Korea; ^2^Catholic Research Institute for Intractable Cardiovascular Disease (CRID), College of Medicine, The Catholic University of Korea, Seoul, South Korea

**Keywords:** postmenopausal women, central blood pressure, cardiovascular disease, blood pressure control, pulse pressure

## Abstract

**Background:**

Hypertension (HBP) is a common disease among both men and women. Central blood pressure (CBP) is a method of evaluating aorta pressure that can assess the intrinsic BP of an individual patient that more closely correlates with cardiovascular disease (CVD) outcomes than peripheral BP parameters. We evaluated gender-specific differences in CBP and optimal target BP based on a composite outcome of CVD, heart failure (HF), and hypertensive complications in patients with HBP.

**Method:**

Patients were enrolled from June 2011 to December 2015 and were followed through December 2019. CBP was measured using radial tonometry. The primary endpoint was a composite outcome.

**Result:**

The median follow-up period for enrolled patients was 6.5 years. Out of a total of 2,115 patients with an average age of 57.9 ± 13.6 years, 266 patients (12.6%) had events of primary end points during the follow-up period. There was no difference in the lowest BP level between men and women in the incidence of CVD. Among the women (49.6%), 78.1% were postmenopausal. In a multivariable Cox proportional hazards model, CBP and systolic BP showed an increase in risk of 10 and 11%, respectively, with every 10 mmHg increase, and there was a similar trend of 12 and 13%, respectively, in postmenopausal women. However, PP showed an increase in risk of about 2% every 10 mmHg increase, but a tendency to increase risk by 19% in postmenopausal women.

**Conclusion:**

This study demonstrated that postmenopausal women will continue to show increased risk for CVD at BP higher than the optimal level. Conversely, there was no increase in CV risk due to menopause at BP values below the optimal level. Therefore, well-controlled BP is more important in postmenopausal women.

## Introduction

Hypertension (HBP) is a common disease occurring in about 30% of male and female adults. That means an estimated 600 million men and women globally have HBP. The prevalence has doubled over the past 30 years, and though the number of well-controlled BP patients has increased significantly, the proportion of controlled patients in all patients with HBP is still small (1). Based on studies conducted by the SPLINT trial (2) and ACCORD trial (3), strict BP control shows better cardiovascular (CV) outcomes, forcing physicians to set stricter target BPs in the treatment of HBP (4). Therefore the importance of obtaining reliable BP readings in patients with hypertension and comorbidities (5). For this reason, the 2017 ACC/AHA Guidelines recommended lowering the existing threshold BP target from 140/90 mmHg to less than 130/80 mmHg (6). As shown in a meta-analysis, the lowest systolic blood pressure (SBP) was less than 115 mmHg (4). In a study using a mercury manometer on more than 370,000 people in Korea, the BP with the lowest hazard ratio was repeatedly found at an SBP of 115 mmHg and a diastolic blood pressure (DBP) of 70 mmHg (7). The sharpest increase in CV events was at an SBP of around 120–140 mmHg and a DBP of 75–90 mmHg, which is a reminder of the need for more stringent BP level recommendations. Central blood pressure (CBP) is a method of evaluating aorta pressure, which can determine the intrinsic BP of an individual patient and more closely correlates with CVD outcomes than peripheral BP parameters (8).

Meanwhile, awareness of the difference in BP between men and women is increasing, and previous studies have reported that the difference in BP between men and women, especially middle-aged and older women, expands at a faster rate than men (9–11). Apart from this awareness of BP, in the process of arterial aging, several studies have shown that the BP curve in adult men continues to increase with age, but in adult women, a sharp increase in BP is observed in connection to menopause (12). However, despite these gender-specific differences in BP, the Lewington et al. (4) study published in 2002 did not report significant CV outcome differences between men and women, and the HYVET study published in 2008 also confirmed that there were no significant differences in CV outcomes by gender (13). In a UK study in which 54% of more than 30,000 of the cohort were women, BP was measured using a mercury sphygmomanometer. Sex-specific analyses indicated that BP measures actually progressed more rapidly in women than in men, beginning early in life. However, the cumulative number of CV events tended to be higher in men (14). Another study in 27,543 patients without a history of cerebrovascular disease followed almost 30 years reported that CVD risk is associated with elevations from lower SBP ranges in women but not in men. As a result, exposures leading to SBP elevation above gender-specific normal ranges may also elevate CVD risk in a gender-specific manner (15).

Therefore, the aim of this study is to investigate the relationship between the optimal CBP and SBP according to the gender of non-invasively measured CBP and SBP using CVD outcomes.

## Methods

### Study population

An initial cohort of participants who presented with or without concomitant CVD risk factors or target organ damage was selected from the Department of Internal Medicine, St. Vincent's Hospital, from July 2011 to December 2015. Among the patients who underwent noninvasive, semiautomated, radial artery applanation tonometry (Omron HEM-9010AI) and were eligible for our study, 2,115 [1,066 (50.4%) male; mean age: 57.9 ± 13.6 years] were enrolled in this study. In addition, female patients were classified as pre or postmenopause through a survey. The postmenopausal women numbered 819 (78.1%). Among the participants, patients with irregular cardiac rhythm who could not undergo radial tonometry or patients with brachial artery stenosis who could not undergo brachial BP measurement were excluded from the experiment. In addition, those who had a CV event within 3 months of the participation date of the experiment were excluded from participation.

There was no industry involvement in the design, implementation, or data analysis of this study. The present study was a single-center retrospective study and was approved by the Institutional Review Board of St. Vincent's Hospital (VC22RISI0070).

### Measurement of brachial blood pressure and central blood pressure

All participants underwent measurement of SBP, DBP, arterial pressure, and pulse pressure (PP) in a comfortable chair after at least 5 min of rest in a quiet room at a constant temperature, and the average of two readings was used. Measurements were taken from the right upper arm using a cuff-oscillometric device (HEM907, Omron Healthcare). At the same time, radial pulse waves were obtained with an automated applanation tonometer (HEM-9010AI). The method used to measure CBP was the same as in a previous study (16).

### Clinical and biochemical assessments

Blood samples were obtained after between 12 and 14 hours of fasting (8:00 p.m. to 9:30 a.m.) to minimize the effect of circadian variation. Total cholesterol concentration followed standard enzymatic methods; high density lipoprotein (HDL) was measured after precipitating very low density lipoprotein (VLDL) and low density lipoprotein (LDL) with phosphotungic acid, and LDL was calculated according to Friedewald's formula. It was the same condition as in our previous studies (15). eGFR calculation was done using the CKD-EPI formula.

### Outcome

The primary endpoint was a composite outcome of atherosclerotic cardiovascular disease (ASCVD) events, including HBP complications or death. ASCVD included other alternative dimensions such as stroke, transient ischemic attack (TIA), and peripheral artery disease (PAD) as well as coronary dimensions such as acute coronary syndrome (ACS) or chronic coronary syndrome (CCS). PAD was set to the ankle to a brachial index (ABI) < 0.9, and Omron VP-1000 Viscular Profiler (Omron Healthcare) was used for measurement. During the follow up period, the patient's medical records were reviewed by a circulatory physician to determine the occurrence of primary endpoints.

### Statistical analysis

Continuous variables were expressed as mean ± standard deviation, and categorical variables were expressed as absolute and relative frequencies. The *t*-test was used to compare the mean between the two groups, and the ratio was tested for two-way tables and chi-square. To determine the independent predictors of the primary endpoints, we used a multivariate analysis employing the Cox proportional risk regression model for a significant risk factor that is significant in the univariate analysis and known for the primary endpoints. Multivariate analysis was plotted using a limited cubic spline curve. All statistical analysis was done with version 3.6.3.

## Results

The median follow-up period for enrolled patients was 6.5 years, with an average age of 57.9 ± 13.6 years, 50.4% for men and 49.6% for women. Among the women, 21.9% were premenopausal, and 78.1% were postmenopausal. Of a total of 2,115 patients, 266 patients (12.6%) had primary end point events during the follow-up period. Among them, eight patients died of cardiac death, 122 were diagnosed with vascular disease including ACS and coronary reperfusion, and 34 experienced stroke, TIA, or brain hemorrhage. The occurrence of heart failure in 21, HBP complications in 62, and atrial fibrillation in 19 was confirmed.

Table 1A shows the baseline characteristics for all participants and by gender. The average age was about 60 years old of female, and 56 years old of male. There were significant differences in history of smoking, FBS and lipid profile except for LDL between male and female. And also, these two groups showed significant differences in CBP, SBP and PP. Table 1B shows baseline characteristics divided by menopause in female participants. The average age of all participating females was about 60 years old; the mean age of premenopausal women was about 41 years old and that of postmenopausal women was 65 years old. Except for age, these two groups did not show significant differences in body mass index (BMI), smoking status, or HbA1c. The value by BP measurements showed significant differences of 126 mmHg for CBP and 134 mmHg for SBP in postmenopausal women, and PP showed a significant difference of more than mean 12.6 mmHg based on menopause state.

Table 1**(A)** Baseline characteristics of the participants. **(B)** Baseline characteristics of the participants divided by menopause.

**Table:** 

	**Overall**	**Female**	**Male**	**P value**
**(A)**				
Variables	2,115	1,049	1,066	
Age, years (SD)	57.92 (13.57)	59.93 (13.61)	55.95 (13.24)	< 0.001
BMI, kg/m^2^ (SD)	24.47 (3.44)	24.41 (3.68)	24.54 (3.20)	0.380
Smoking, N (%)	577 (28.1)	41 (4.0)	536 (51.6)	< 0.001
FBS, mg/dl (SD)	119.38 (46.78)	114.81 (41.32)	123.87 (51.23)	< 0.001
HbA1c, % (SD)	7.01 (1.79)	7.07 (1.74)	6.97 (1.82)	0.468
TC, mg/dl (SD)	186.13 (42.16)	189.62 (41.53)	182.76 (42.50)	0.001
TG, mg/dl (SD)	135.21 (101.33)	124.19 (87.64)	145.89 (112.03)	< 0.001
LDL, mg/dl (SD)	111.22 (35.77)	112.40 (36.05)	110.13 (35.48)	0.204
HDL, mg/dl (SD)	43.94 (11.58)	46.64 (11.81)	41.44 (10.78)	< 0.001
CBP, mmHg (SD)	121.25.12 (19.77)	124.26 (20.48)	118.31 (18.60)	< 0.001
SBP, mmHg (SD)	131.67 (18.82)	132.25 (19.71)	131.09 (17.88)	0.042
DBP, mmHg (SD)	77.58 (12.52)	77.09 (12.19)	78.06 (12.82)	0.874
PP, mmHg (SD)	54.13 (14.27)	55.19 (15.46)	53.08 (12.91)	0.006
AIx@75, (SD)	81.82 (15.34)	87.59 (13.79)	76.15 (14.67)	0.163
Heart Rate, (SD)	72.45 (12.54)	73.10 (13.03)	71.82 (12.01)	0.775

**Table:** 

**(B)**			
	**Premenaupausal**	**Postmenaupausal**	**P value**
Variables	230	819	
Age, years (SD)	40.80 (7.77)	65.30 (9.43)	< 0.001
BMI, kg/m^2^ (SD)	24.35 (4.45)	24.42 (3.43)	0.789
Smoking, N (%)	11 (5.0)	30 (3.8)	0.548
FBS, mg/dl (SD)	106.81 (32.58)	116.76 (42.98)	0.004
HbA1c, % (SD)	7.12 (2.25)	7.07 (1.66)	0.883
TC, mg/dl (SD)	193.22 (37.50)	188.81 (42.37)	0.234
TG, mg/dl (SD)	124.84 (105.00)	124.04 (83.25)	0.918
LDL, mg/dl (SD)	117.37 (31.99)	111.34 (36.80)	0.076
HDL, mg/dl (SD)	48.31 (13.53)	46.27 (11.37)	0.066
CBP, mmHg (SD)	118.00 (20.25)	126.02 (20.21)	< 0.001
SBP, mmHg (SD)	126.10 (19.56)	133.98 (19.42)	< 0.001
DBP, mmHg (SD)	80.76 (13.43)	76.05 (11.61)	< 0.001
PP, mmHg (SD)	45.32 (11.25)	57.96 (15.35)	< 0.001
AIx@75, (SD)	84.45 (12.61)	87.42 (11.03)	0.001
Heart Rate, (SD)	77.48 (14.53)	73.24 (12.98)	< 0.001

Alx@75, Augmentation index adjusted with a heart rate of 75; BMI, Body mass index; CI, Confidential interval; eGFR, estimated Glomerular filtration rate; TC, Total cholesterol; TG, Triglyceride; LDL, Low densitiy lipoprotein; HDL, high density lipoprotein; CBP, Central blood pressure; SBP, Systolic blood pressure; DBP, Diastolic blood pressure; PP, Pulse pressure.

Table 2 presents the results of univariate Cox proportional hazard ratio models for each variable in men and women before and after menopause. In postmenopausal women, CBP and SBP showed a risk increase of 13 and 15%, respectively, for every 10 mmHg increase, and PP also showed a risk increase of 25% for every 10 mmHg. On the other hand, in men, the risk increase was about 7% with the increase of CBP 10 mmHg, but it was not statistically significant, but in SBP, the 10% risk increase with the increase of 10 mmHg was confirmed at a statistically significant level. In addition, PP also showed a 17% increase with an increase of 10 mmHg.

**Table 2:** Univariate Cox proportional hazards model.

	**Male**		**Premenopausal**		**Postmenopausal**	
**Events/N**	**157/1,066**		**7/230**		**102/819**	
**Variables**	**Hazard ratio**	**P value**	**Hazard ratio**	***P*** **-value**	**Hazard ratio**	**P value**
BMI, kg/m^2^	1.01 (0.95–1.05)	0.971	0.97 (0.82–1.14)	0.688	1.04 (0.98–1.10)	0.227
HbA1c, %	0.95 (0.83–1.09)	0.463	0.17 (0.01–3.74)	0.258	1.01 (0.84–1.20)	0.979
eGFR, mL/min/1.73 m^2^	0.98 (0.97–0.99)	< 0.001	0.98 (0.95–1.01)	0.181	0.99 (0.98–1.00)	0.059
TC, mg/dl	0.99 (0.99–1.00)	0.133	1.01 (0.99–1.02)	0.628	0.99 (0.99–1.00)	0.137
LDL, mg/dl	0.99 (0.99–1.00)	0.325	1.01 (0.99–1.03)	0.484	0.99 (0.99–1.00)	0.036
HDL, mg/dl	0.98 (0.96–1.00)	0.015	0.98 (0.93–1.04)	0.505	0.97 (0.95–0.99)	0.014
CBP, per 10 mmHg	1.07 (0.99–1.16)	0.069	0.95 (0.69–1.30)	0.740	1.13 (1.04–1.24)	0.004
SBP, per 10 mmHg	1.10 (1.01–1.19)	0.028	0.87 (0.60–1.27)	0.472	1.15 (1.05–1.27)	0.003
DBP, per 10 mmHg	0.89 (0.78–1.00)	0.059	0.91 (0.55–1.51)	0.722	0.93 (0.79–1.10)	0.416
PP, per 10 mmHg	1.28 (1.15–1.42)	< 0.001	1.02 (0.61–1.69)	0.950	1.25 (1.12–1.41)	< 0.001
AIx@75	1.17 (1.04–1.32)	0.01	1.04 (0.61–1.790)	0.874	0.95 (0.80–1.13)	0.560
Heart Rate,	1.03 (0.91–1.17)	0.623	0.89 (0.53–1.48)	0.650	1.02 (0.86–1.21)	0.836

Alx@75, Augmentation index adjusted with a heart rate of 75; BMI, Body mass index; CI, Confidential interval; eGFR, estimated Glomerular filtration rate; TC, Total cholesterol; TG, Triglyceride; LDL, Low densitiy lipoprotein; HDL, high density lipoprotein; CBP, Central blood pressure; SBP, Systolic blood pressure; DBP, Diastolic blood pressure; PP, Pulse pressure.

Table 3 showed a multivariable Cox proportional hazards model adjusted by age and BMI. In postmenopausal women, CBP, SBP, and PP for every 10 mmHg increased the risk by 12, 13, and 19%, respectively and in overall participants 10, 11, and 2%, respectively.

**Table 3:** Multivariate Cox proportional hazards model.

	**Overall**		**Male**		**Premenopausal**		**Postmenopausal**	
**Variables**	**Hazard ratio** **(95% CI)**	**P value**	**Hazard ratio** **(95% CI)**	**P value**	**Hazard ratio** **(95% CI)**	**P value**	**Hazard ratio** **(95% CI)**	**P value**
CBP, per 10 mmHg	1.10 (1.01–1.20)	0.021	1.04 (0.96–1.12)	0.34	0.93 (0.68–1.27)	0.64	1.12 (1.02–1.22)	0.01
SBP, per 10 mmHg	1.11 (1.01–1.22)	0.029	1.08 (0.99–1.18)	0.08	0.87 (0.61–1.24)	0.45	1.13 (1.03–1.25)	0.01
DBP, per 10 mmHg	0.99 (0.84–1.17)	0.911	1.00 (0.87–1.14)	0.91	0.89 (0.53–1.49)	0.66	0.99 (0.83–1.18)	0.92
PP, per 10 mmHg	1.02 (1.00–1.03)	0.010	1.01 (1.00–1.03)	0.010	0.97 (0.57–1.67)	0.92	1.19 (1.05–1.35)	0.01
AIx@75	0.96 (0.81–1.13)	0.615	1.05 (0.92–1.20)	0.490	0.97 (0.53–1.77)	0.93	0.96 (0.80–1.14)	0.61
Heart Rate	0.99 (0.84–1.17)	0.917	1.08 (0.95−1.23)	0.250	0.89 (0.53–1.51)	0.67	1.01 (0.84–1.20)	0.99

Alx@75, Augmentation index adjusted with a heart rate of 75; CBP, Central blood pressure; CI, Confidential interval; SBP, Systolic blood pressure; DBP, Diastolic blood pressure; PP, Pulse pressure.

**Figure 1:**
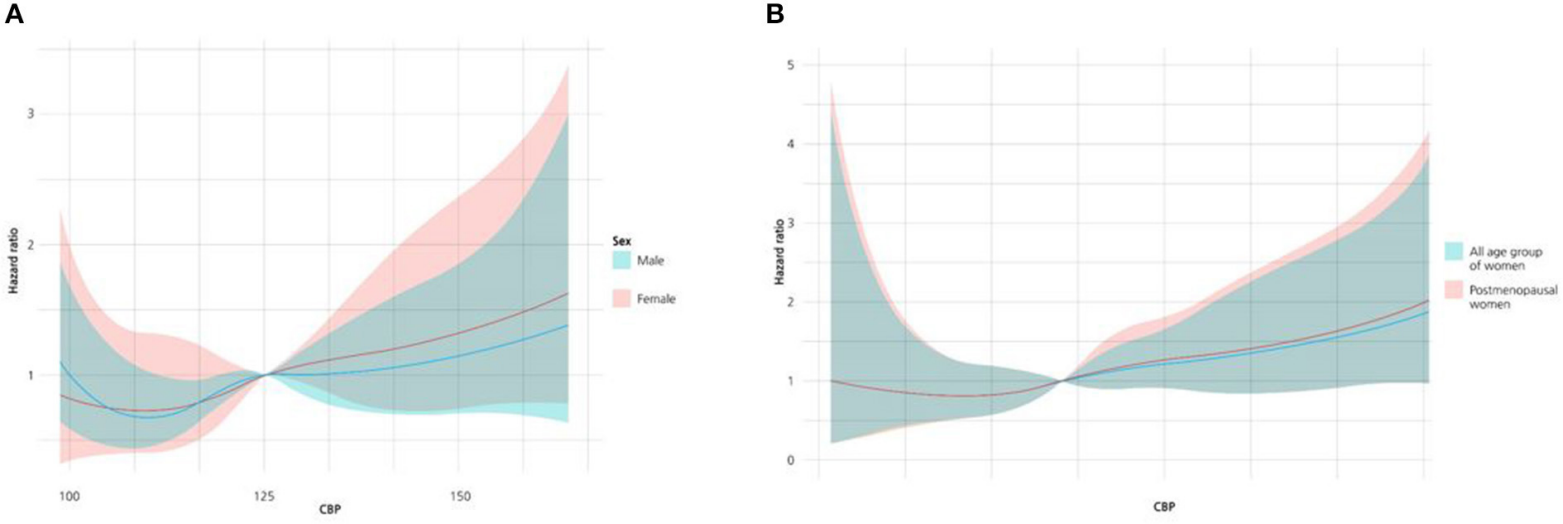
**(A)** Restricted cubic spline curve of cumulative incidence of the primary outcome according to the entire age group of men (red) and women (blue). **(B)** Restricted cubic spline curve of cumulative incidence of the primary outcome according to all age group of women (blue) and group of postmenopausal women (red).

In Figure 1A, the hazard ratio according to CBP increase in all age groups of men and women was analyzed with a restrictive cubic spline curve. The lowest level of CBP (115 mmHg) with the lowest hazard ratio was not different between men and women, and the risk showed a J-shaped pattern. In addition, the point thought to be optimal CBP was no different between men and women (126 mmHg). In Figure 1B, the hazard ratio pattern according to CBP increase was compared for all age groups of women and the postmenopausal age group, and it was confirmed that the hazard ratio was consistently higher in postmenopausal women than the all-age group after passing the optimal point (126 mmHg).

## Discussion

This study found that CVD risk according to CBP in both men and women had a J-shaped curve, and there was no difference between men and women at the lowest incidence of primary endpoints. The lowest levels of CBP and SBP were 115 and 126 mmHg, respectively. However, at higher BP than the lowest BP level, the increase in the CV risk due to increasing BP was more rapid in women than in men, and this difference increased as BP increased.

Similar to our findings, a meta-analysis study reported no difference in optimal BP levels for CVD risk between men and women in peripheral BP (17). Also, in the SPRINT trial, there was no difference in the threshold level according to the gender (2). In addition, there were several large-scale studies showing a J-shaped curve in peripheral BP parameters and CVD risk. As confirmed in large-scale studies, women over a certain age show a steeper slope compared to men (14, 18).

In our study, we used CBP to better understand individual BP conditions rather than measuring peripheral BP (19). We found that the risk of CVD with increasing CBP was consistently steeper at the above optimal level in postmenopausal women than in an all-age female group. We developed several hypotheses about this rapid rise in BP in postmenopausal women. The first hypothesis is related to hormonal changes in postmenopausal women. The evidence suggests that early postmenopausal women taking hormone replacement therapy (HRT) have a reduced risk of coronary artery disease (20, 21). However, studies have shown the contrary, so this is still a controversial hypothesis (22). Regarding this increase in BP and CVD risk in postmenopausal women, research tends to focus on pathophysiological characteristics different from those of men caused by hormonal changes rather than the hormones themselves. Hormonal changes with menopause result in changes in endothelial vasodilation, nitric oxide pathways, sympathetic and renin-angiotensin-aldosterone system endothelin production, and these changes affect aortic stiffness in older women. This may lead to greater BP variability (BPV) in women than in men (23, 24). Increased BPV results in greater mechanical stress on the arterial wall, leading to adverse structural changes within the arterial wall. These changes have been shown to play an important role in aortic stiffness (25). Aortic stiffness may be closely related to increased risk of CVD (26).

Another finding in our study was that larger differences in PP in postmenopausal women resulted in more CV events, but a similar J-shaped pattern to BP in men. In the case of men, CV events occurred more frequently in the very low PP section, and other studies showed similar results (27).

Our study also investigated participants' use of antihypertensive drugs. As a result, out of 230 premenopausal women, 63 were using antihypertensive drugs (including duplicate use), among which angiotensin-converting enzyme inhibitor (ACEi) or angiotensin II receptor blocker (ARB) was 33 (14%) beta-blocker was 14 (6%) calcium channel blocker (CCB) was 42 (18%) and diuretics were 13 (6%) was investigated. In addition, among 819 postmenopausal women, 385 were using antihypertensive drugs (including duplicate use), among which ACEi or ARB was 223 (27%) beta-blocker was 127 (16%) CCB was 171 (21%) and diuretics were 119 (15%).

However, as there were unclear points such as the time of taking the drug and compliance, no adjustment was made during statistical analysis, which is suggested as a limitation of our study. And there are other limitations as to whether or not HRT. In our study, the subjects notified self-reported survey whether it was menopause at the measurement of CBP. Simultaneously, hormone replacement therapy was checked and excluded. However, we did not investigate anymore whether HRT was used during follow up period. In addition, in menopause women, the menopause period at the time of CBP measurement was not investigated and could not be corrected during statistical analysis. Female hormones are an important factor that can affect our results, which is thought to be a limitation.

## Conclusion

This study demonstrated that both men and women exhibited a J-shaped pattern in the incidence of CBP and CV risk and showed that there was no difference in BP between men and women in the lowest incidence of CVD. However, postmenopausal women continued to show an increased risk for BP higher than the optimal level. Conversely, CV risk due to menopause was not evident at BP below the optimal level. This means that BP control is more important in postmenopausal women. However, further studies are needed to determine the pathophysiological and clinical causes of rapid changes in BP in postmenopausal women in the future.

## Data availability statement

The raw data supporting the conclusions of this article will be made available by the authors, without undue reservation.

## Ethics statement

The studies involving human participants were reviewed and approved by VC22RISI0070. Written informed consent for participation was not required for this study in accordance with the national legislation and the institutional requirements.

## Author contributions

M-SK and G-HK participated in the study design, data collection, and performed the statistical analysis and drafted the article. Both authors contributed to the article and approved the submitted version.

## Conflict of interest

The authors declare that the research was conducted in the absence of any commercial or financial relationships that could be construed as a potential conflict of interest.

## Publisher's note

All claims expressed in this article are solely those of the authors and do not necessarily represent those of their affiliated organizations, or those of the publisher, the editors and the reviewers. Any product that may be evaluated in this article, or claim that may be made by its manufacturer, is not guaranteed or endorsed by the publisher.

## Abbreviations

ABI: Ankle to brachial index
ACS: Acute coronary syndrome
(ACEi): Angiotensin-converting enzyme inhibitor
(ARB): Angiotensin II receptor blocker
ASCVD: Atherosclerotic cardiovascular disease
ASO: Arteriosclerosis obliterans
BP: Blood pressure
BPV: Blood pressure variability
BMI: Body mass index
(CCB): Calcium channel blocker
CCS: Chronic coronary syndrome
CV: Cardiovascular
CBP: Central blood pressure
CVD: Cardiovascular disease
CI: Confidence interval
DBP: Diastolic blood pressure
HF: Heart failure
HBP: Hypertension
HDL: High-density lipoprotein
HRT: Hormone replacement therapy
LDL: Low-density lipoproteins
MI: Myocardial infarction
PAD: Peripheral arterial disease
PP: Pulse pressure
SBP: Systolic blood pressure
TIA: Transient ischemic attack
VLDL: Very low density lipoprotein.
